# Strengthening Africa’s resilience to Mpox: Preparedness and response initiatives of the Pasteur network

**DOI:** 10.4102/jphia.v16i1.1026

**Published:** 2025-03-31

**Authors:** Delia D. Djuicy, Benjamin Selekon, Christian Malaka, Aboubacar Soumah, Edgar Adjogoua, Hervé Kadjo, Offianan A. Toure, Pierre Roques, Solène Grayo, Noël Tordo, Ousmane Faye, Abdourahmane Sow, François-Xavier Berthet, Vincent Lacoste, Soa Fy Andriamandimby, Hamidou L. Ramatoulaye, Adamou Lagare, Haoua S. Sabo, Ella Farra, Sandra G. Ouangole, Gide Martial Yonga Wansi, Dieudonne G. Essima, Jules Landry Mounchili Mouliem, Herman Philipe Nfombouot Njitoyap, Sandrine O. Nkoum Kyane, Syndou Meite, Sara I. Eyangoh, Mirdad Kazanji, Yap Boum, Rebecca Grais, Emmanuel Nakoune, Richard Njouom, Amadou A. Sall

**Affiliations:** 1 Centre Pasteur du Cameroun, Yaoundé, Cameroon; 2 Institut Pasteur de Bangui, Bangui, Central African Republic; 3 Institut Pasteur de Côte d’Ivoire, Abidjan, Côte d’Ivoire; 4 Institut Pasteur de Guinée, Conakry, Guinea; 5 Institut Pasteur de Dakar, Dakar, Senegal; 6 Institut Pasteur de Madagascar, Antananarivo, Madagascar; 7 Centre de Recherche Médicale et Sanitaire (CERMES), Niamey, Niger; 8 Pasteur Network, Paris, France; 9 School of Health Sciences, Institut Pasteur de Bangui, Bangui, Central African Republic

## Introduction

Mpox is a major public health concern in Africa and is recognised as a recurring and current public health emergency. Although globally less serious than smallpox, which was eradicated in the 1980s, this disease poses a serious threat to global health, because of its potential for rapid spread and its association with morbidities and mortality.^1^

Mpox is a zoonotic viral disease caused by the mpox virus (MPXV). Mpox virus has two distinct lineages: the ancient Congo Basin Clade I, endemic to Central Africa, and the ancient West African Clade II prevalent in West Africa.^2^ Clade II is further split into Clade IIa and Clade IIb. Before 2022, less than a hundred of cases were reported outside Africa; all associated to exotic pet trade and international travels connected to Ghana and Nigeria.^3,4,5,6,7,8^ The high number of reported cases caused by Clade IIb viruses out of Africa led to mpox being declared a Public Health Emergency of International Concern (PHEIC) by the World Health Organization (WHO) on 23 July 2022. The outbreak was declared over 1.5 years later.^9^

A Public Health Emergency of Continental Security (PHECS) was declared by the Africa Centre for Disease Control and Prevention (Africa CDC) on 13 August 2024 and a new PHEIC was declared by the WHO on 14 August 2024 because of increasing number of cases in Africa and the reporting of new cases in naïve areas.^9^ This time however, the PHEIC and PHECS are associated with the emergence of a new viral strain of the Clade I leading to the split of the Clade into two subclades: Clade Ia and Clade Ib.^10,11^ Both subclades originate from the virus responsible for outbreaks in Central Africa. Clade Ia encompasses all MPXV Clade I strains ever described while Clade Ib is a group of new genetically distinct variants circulating in Eastern Democratic Republic of Congo (DRC) and Eastern Africa. The factors that led to the emergence of Clade IIb remains unknown.

A total of 11 African countries are historically endemic for mpox: Benin, Cameroon, the Central African Republic (CAR), the DRC, Gabon, Côte d’Ivoire, Liberia, Nigeria, the Republic of Congo, Sierra Leone and South Sudan. In these countries, the number of reported mpox cases has been increasing over decades: 48, 357 and 520 cases were reported in the 1970s, 1980s and 1990s, respectively.^1,12^ The firsts two decades of the 2000s saw a sharp increase in cases, with over 10 000 and 20 000 cases reported respectively. The DRC accounted for 95% of these cases.^1^

Throughout 2023, Africa recorded 14 838 mpox cases (1665 confirmed and 13 173 suspected) across seven countries, with 738 confirmed deaths.^9^ The situation has evolved since the beginning of 2024, with 18 737 mpox reported cases (3101 confirmed and 15 636 suspected) across 12 African countries by 16 August 2024.^9^ The current Clade Ib outbreak has already expanded to CAR and eastern African countries including Rwanda, Burundi, Uganda and Kenya; while imported cases were identified in Sweden and Pakistan.^8^ Historically, reported case fatality rates (CFRs) differ significantly between Clade I (> 10%) and Clade II (1% – 5%).^12,13^ The Clade Ib, however, has greater transmissibility and potential for more severe clinical outcome.^9^

## Preparedness and response to mpox of the Pasteur Network in Africa

The Pasteur Network (PN), and in particular the institutes based in the Africa region (PN-Africa), are concerned about the evolution of mpox in the sub-region. The substantial upsurge in mpox cases, and spread beyond Africa, clearly points to the urgent need for prompt and decisive action to contain and eliminate mpox as a public health threat. Pasteur Network-Africa has therefore taken on the urgent mission of advocating and contributing to the implementation of a coordinated and integrated approach combining prevention, diagnosis, treatment, vaccination and public awareness to better tackle the disease throughout the African region. In this perspective, we provide updates on the outbreak in each member state as of 31 October 2024.

In *Cameroon*, the Centre Pasteur du Cameroun (CPC), which hosts the National Reference Laboratory (NRL) for mpox surveillance, is pivotal to preparedness and response activities aimed at controlling the outbreak.^14^ The first mpox case in Cameroon was reported in 1979 in a village located in the Centre region 40 km from Yaoundé, the capital city.^15^ On 30 August 2024, 278 mpox suspected cases have been identified by the national surveillance programme since the first identified case, and 65 have been laboratory-confirmed.^16^ Up to 2018, only three sporadic human cases were cumulatively confirmed for MPXV infection, in 1979, 1980 and 1989.^15,16,17,18,19^ This was followed by a 30-year pause with no reported case in humans. However, two epizootics occurred in captive chimpanzees in 2014 and 2016.^20^

A change in the trend was observed between 2018 and 2024 with a continuous increase in the number of laboratory-confirmed mpox cases: from only 1 confirmed case among 29 suspected cases in 2018, 1 confirmed case in 2019 among 7 suspected cases,^15,16^ 5 confirmed cases in 2020 and 2021 respectively, and 17 confirmed among 84 suspected cases in 2022.^16^ The highest positivity rate occurred in 2023 with 28 confirmed cases out of 99 suspected cases. The latest data from 2024 indicate 6 confirmed cases out of 92 suspected cases as of 31 October 2024.

In Cameroon mpox is reported only in the rural forested areas in the southern part of the country. The circulation of both Clade I and Clade II MPXV is a unique feature of mpox epidemiology in the country. However, genomic surveillance reports a clear geographic separation of these two clades; Clade I is prevalent in eastern regions close to neighbouring mpox-endemic countries of Central Africa while Clade II is found in western regions close to West Africa.^16,21^

The suspicion is that the re-emergence of MPXV after a 30-year lapse is probably from a different viral reservoirs unique to the specific ecosystems of eastern and western rainforests.^16^ This history of mpox in Cameroon has led the NRL in the country to develop expertise in MPXV detection,^14,15,16,21^ and is supporting members of the PN with the diagnosis and genomic monitoring of circulating and emerging MPXV strains.

In the *Central African Republic* (*CAR*), the Institut Pasteur de Bangui (IPB) also hosts the NRL for mpox surveillance for the country. The first micro-outbreak involving four confirmed cases occurred in 1984 in Lidjombo (Sangha Mbaéré prefecture) in the extreme southwestern region of the country.^22^ The next four cases were identified in 2001 in the Bangassou Health District (Mbomou prefecture), about 800 km from Bangui on the banks of the River Oubangui, which borders the DRC.^23^ From 2001 to 30 August 2024, the Department for Epidemiological surveillance of the Ministry of Public health (MoH) in CAR has recorded 1077 suspected cases of mpox of which 182 cases were molecularly confirmed as MPXV infection by the IPB.

This evidence of mpox circulation in CAR evolved slowly, first with no detected cases between 2001 and 2009, then four cases identified between 2010 and 2015. The sporadic nature of these four cases suggested the absence of a systematic surveillance for the disease.^24^ Active surveillance for mpox in CAR began in 2016 and continues today with laboratory-confirmed cases reported every year: seven cases were recorded in 2016 and six cases were recorded in 2017. The annual reporting rate increased significantly in 2018 with 27 confirmed cases, but fell to 15 confirmed cases in 2019^24,25^ In 2020, during the coronavirus disease 2019 (COVID-19) pandemic, 9 cases were reported. The number of confirmed cases rose again to 28 in 2021, 17 in 2022, and 19 in 2023.^25^ By the 31 October 2024, 66 of the 321 suspect cases had been confirmed.

Most confirmed cases occurred in the forested and rural areas of the south-eastern districts along the Oubangui River, which is a long border with the DRC. In 2024, marked by the emergence of an urban epidemic, nine confirmed cases were identified in Bangui, the capital city of the CAR.

Sequencing data from 2001 to 2018 showed that three lineages of MPXV were circulating in CAR, all closely related to Clade Ia strains found in the DRC and the global tropical forest of the Congo Basin.^24^ The viral strains from the most recent epidemics in 2023 and 2024 are from Clade Ia with no association with the current Ib variant.

The resurgence of the MPXV in the CAR could be linked to changes in the ecosystem caused by deforestation and subsistence hunting.^24,25^ The IPB plays a key role in the response to mpox epidemics in CAR, detecting all suspected cases and defining the circulating strains, to help the MoH undertake appropriate measures to control the spread of the disease.

In *Cote D’Ivoire*, the Institut Pasteur of Côte d’Ivoire (IPCI) is the national reference centre responsible for mpox diagnostic in the country. The first two human mpox cases were reported in 1971 in the Bossematié forest in Abengourou in the region of Indénié-Djuablin, in the eastern part of the country close to Ghana. The human case reported in 1981 originated in a village in Daloa in the Haut-Sassandra region in west-central Côte d’Ivoire.^23^ There was no other human case reported in the country until 03 July 2024 when the index case for the country’s outbreak was confirmed by the IPCI. Until then, there was a fatal case in a chimpanzee confirmed in the Tai forest in the western part of the country in 2012.^26^

The confirmed index case for the 2024 outbreak in the country was a 46-year-old patient, living in a rural area of Iboke in the west side of the country, in the Tabou department of the Bas-Sassandra region. The patient reported contact with rodents. The number of cases had increased to 402 suspected cases of which 89 cases were confirmed, and one death reported by 31 October 2024. In addition, 28 districts are affected mostly located in the western and eastern part of the country.

The virus is circulating in both rural and urban areas. More men (65.4%) than women (37.4%) have confirmed infection. The majority of infected individuals are 15 to 39 years old. Pupils and students (28%), and farmers and hunters (16%) seems to be the most affected populations. The West African Clade IIa was identified in 31.6% of confirmed cases. One confirmed case was infected with Clade IIb.

Côte d’Ivoire is facing its first major outbreak of the mpox epidemic. The epidemiological link in the current outbreak is difficult to establish, and requires a One Health multidisciplinary approach to understand the emergence of the virus in the country. Since the first case was reported in early July 2024, an active surveillance has been instituted through the Public Health Emergency Operations Center (COUSP) in response to the outbreak. The IPCI is collaborating with the COUSP and the MoH to curb the outbreak in the country.

In *Guinea*, the Institut Pasteur de Guinée (IPGui) is involved in the survey of emerging diseases through a collaborative network with other Guinean laboratories. IPGui has established a molecular diagnostic method to distinguish mpox from chickenpox during the 2022 PHEIC. In addition, building on the expertise on viruses responsible for rashes and skin lesions, the IPGui is developing a molecular differential diagnosis for mpox, Varicella Zooster Virus (VZV), Human Herpes virus (HHV) type 1–2, HHV6, parvovirus B19, measles, rubella and enteroviruses involved in hand-foot-and-mouth disease. This will provide a more comprehensive view of the possible aetiologies associated with the various skin lesions seen in Guinea. The molecular diagnostic method to distinguish mpox from chickenpox differential diagnosis has been used to demonstrate the circulation of several cases of the VZV responsible for chickenpox in patients presenting clinical signs mimicking mpox.

Despite circulation of mpox in neighbouring surrounding countries for several years,^27,28,29^ the first case of mpox in Guinea was identified on 30 August 2024 in a 7-year-old child in Komaya-Macenta (Forest Guinea). Before this date, nine suspected cases had been investigated since the beginning of the year, and mpox was ruled-out in all the cases. Since the index case, 51 mpox suspected cases have been identified in Guineas as of 31 October 2024 and only one was confirmed for the MPXV infection. However, 50% of the 26 samples shipped to IPGui were confirmed to be VZV. The confirmed MPXV case had a Clade IIa infection linked to infection strains reported in Liberia in August 2024 and in Sierra-Leone in 2014–2017.

The suggestive evidence is that MPXV re-emerged in the country in early/late 2020/2010 after a 45-year lapse. It probably arose from viral reservoirs unique to ecosystems in south rainforests shared by Guinea, Sierra Leone, Liberia and Cote D’Ivoire.^29^ The current mpox outbreak in Guinea is not critical, though surveillance and emergency preparedness response are in place.^30^

*Other countries*: The other Institute of the PN-Africa located in sub-Saharan Africa are the Institut Pasteur de Madagascar (IPM), Institut Pasteur de Dakar (IPD) in Senegal, and the Centre de Recherche Médicale et Sanitaire (CERMES) in Niger. These institutes are key actors in the disease surveillance in their respective countries in close collaboration with the countries’ Ministry of Health. Even though these countries have not made a diagnosis of mpox, there is regular assessment of suspected cases because of the high-risk for MPXV importation during the ongoing outbreak.

For example, the IPD hosts the NRL for mpox surveillance and plays a pivotal role in the disease preparedness and surveillance in Senegal and the West African Region. Institut Pasteur de Dakar started mpox surveillance in 2017, holding a sequencing platform with a capacity of production of 5000 sequences a week and has tested 251 mpox suspected cases to date. These tests were conducted for samples received from Nigeria (29 cases for in 2017), Côte d’Ivoire (three cases in 2017), Benin (three cases in 2022) and Côte d’Ivoire (two cases in 2024). The institute detected 37 laboratory-confirmed cases from these samples. The institute regularly support Nigeria and Cote D’Ivoire to sequence some of their MPXV strains. The institute regularly host next-generation sequencing (NGS) training workshops for the sub-region and is therefore ready to support members of the PN-Africa in the diagnosis and genomic monitoring of mpox. There is no report of confirmed mpox case in Senegal to date.

Le Centre de Recherche Médicale et Sanitaire (CERMES), the national reference laboratory for viral haemorrhagic fever and zoonosis in Niger, oversees mpox diagnosis and plays a key role in the development of the pandemic emergency plan developed to be prepared for a potential outbreak.

The IPM is similarly equipped with the appropriate molecular tools for mpox diagnosis. The institute has not yet implemented genomic surveillance but will be able to do it in case of emergency with the NGS facility available on site.

## Discussion

The analysis of the reports on the mpox surveillance and response activities going on across African countries reveals critical patterns, gaps and opportunities for action. Key findings emphasise the need to strengthen and expand surveillance systems, enhance laboratory capacities and adopt a multidisciplinary One Health approach to address ecological drivers of the disease. For example, gaps in early detection, such as those seen in CAR and Guinea, highlight the urgency of establishing robust surveillance networks, including genomic monitoring, to track viral evolution and clade-specific distributions.

Ecological changes like deforestation and hunting, linked to mpox re-emergence, underscore the importance of integrating wildlife monitoring into health frameworks. Diagnostic improvements, modelled on Guinea’s distinction of mpox from VZV, alongside regional laboratory collaborations, are recommended to enhance outbreak detection and response.

Community engagement remains pivotal, with tailored public awareness campaigns and the involvement of local leaders in rural and high-risk areas. Strengthening preparedness mechanisms, such as PHECS, PHEIC and rapid response teams, and fostering cross-border collaboration are vital to addressing mpox’s regional and transboundary nature, as demonstrated by its spread along the DRC-CAR border.

Investments in research to explore mpox epidemiology and the development of local vaccines and therapeutics will address emerging transmission trends and reduce dependency on external supplies. Additionally, surveillance must extend to urban centres, where outbreaks pose unique challenges.

In African countries hosting member institutes of the PN, and where residual and imported risk have been identified, mpox surveillance and diagnosis are challenging and vaccines are not available. Vaccination had proven to be an effective tool for the control of smallpox in the last century, band for the control of mpox of 2022 outbreak as shown by the vanishing of Clade IIb multi-country.^1^ Vaccination for the prevention and control of mpox is of critical importance giving the large-scale impact it makes in containing past outbreaks. Vaccination against mpox is particularly relevant for resource-constrained countries hosting the PN-Africa Institutes, where the price of antivirals is unaffordable. In addition, the case for upscaling access to vaccine is strengthened by the absence of an effective treatment, which is likely to be very expensive when developed out of Africa.^31^

## Conclusion

Pasteur Network-Africa proposes a comprehensive and tailored containment plan for regions affected by or at risk of mpox emergence. The strategy integrates advanced tools and methodologies to address the epidemic within a One Health framework. Key components include the deployment of enhanced molecular and rapid diagnostic tools for early detection, genomic surveillance using NGS to monitor viral evolution and viral reservoir characterisation to elucidate spillover events. To further strengthen the response, PN-Africa will incorporate predictive modelling to identify hotspots, anticipate outbreak dynamics, and optimise resource allocation.

The plan also prioritises clinical trials for therapeutics and vaccines to enhance efficacy and foster regional vaccine self-reliance. In addition, it underscores the importance of training diverse stakeholders, strengthening health systems and building technical capacity to ensure a sustainable and effective response.

As an advocate and coordinator, PN-Africa is committed to supporting local initiatives, fostering collaboration with public health authorities, WHO, and Africa CDC, and driving community engagement to ensure inclusivity. By leveraging these tools and partnerships, PN-Africa aims to deliver a robust and scalable response to mpox, safeguarding both regional and global health.
